# Loneliness among refugees: a neglected crisis in need of scalable innovation

**DOI:** 10.1016/j.lanepe.2025.101355

**Published:** 2025-06-17

**Authors:** Shuyan Liu, Daniel J. Bromberg, Matthias Haucke, Xin Zhou, Danielle Poole, Till Bärnighausen, Frederick L. Altice

**Affiliations:** a Department of Psychiatry and Psychotherapy (Campus Charité Mitte), Charité – Universitätsmedizin Berlin, Berlin, Germany; b German Center for Mental Health (DZPG), Germany; c Heidelberg Institute of Global Health (HIGH), Heidelberg University, Heidelberg, Germany; d Yale University School of Medicine, New Haven, CT, USA; e Yale School of Public Health, New Haven, CT, USA; f Africa Health Research Institute, South Africa; g Harvard Center for Population and Development Studies, Cambridge, MA, USA; h APT Foundation, New Haven, CT, USA; i Center for Interdisciplinary Research on AIDS (CIRA), Yale University New Haven, CT, USA

Loneliness is increasingly recognized as a critical determinant of health, with the U.S. Surgeon General recently declaring it a public health crisis, comparable in impact to smoking and excessive alcohol consumption.^1^ While loneliness is widespread globally, it is particularly pervasive among forcibly displaced populations. Refugees fleeing conflict, persecution, or disaster are often separated from family and social networks, placed in unfamiliar environments, and face linguistic, cultural, and structural barriers to integration.^2^^,^^3^ These conditions place them at heightened risk for persistent loneliness, with cascading effects on physical and mental health.

A systematic review showed that up to 50% of refugees and asylum seekers experience loneliness.^3^ This need remains mostly unmet, because few interventions to fight loneliness in this population group have been developed and tested, and the few evidence-based interventions that do exist are rarely implemented. Loneliness among refugees is not simply a symptom of displacement—it is a driver of poor health outcomes, including depression, anxiety, substance use, dementia, cardiovascular disease, social exclusion, and increased all-cause mortality.^1^ Yet global health strategies often neglect social connection in their efforts to improve refugee well-being.

The war in Ukraine has amplified this challenge on a massive scale, with over 6.3 million Ukrainians now displaced across Europe by 2025, compared to 6.8 million globally. Before the conflict, Ukraine already had one of the world's highest loneliness rates. Displacement has exacerbated this, as Ukrainian refugees face the same challenges as other refugee populations: fractured familial and social ties, difficulty accessing health services, and prolonged uncertainty about the future. While this context is urgent and visible, it mirrors patterns observed among Syrian, Afghan, Venezuelan, and Rohingya refugees in prior years and in other global contexts.

Interventions to address loneliness must be low-cost, scalable, and adaptable to the complex realities of mobile, under-resourced populations. Traditional psychosocial supports like group therapy or cognitive behavioural therapy (CBT), while effective, are often impractical in refugee contexts. CBT requires trained providers, cultural adaptation, and long-term engagement that may not align with the transient and resource-limited nature of refugee settings.

In contrast, cognitive reappraisal, a core element of CBT, offers a promising and accessible alternative. It focuses on helping individuals change how they interpret emotionally challenging situations.^4^ Emerging evidence suggests that brief interventions using cognitive reappraisal can reduce loneliness and improve emotional regulation.^5^ These interventions are easier to scale, do not require in-person delivery, and can be integrated to into digital platforms.

Simple behaviour-based interventions such as exercise prompts also show promise. Structured exercise (e.g., yoga and dancing) and everyday non-exercise activities (e.g., taking the stairs and walking) can improve mood, reduce stress, and foster social interaction.^6^ These interventions are cost-effective and can be delivered through mobile devices or group or peer-led sessions in refugee shelters. Their appeal lies in minimal infrastructure needs and adaptability across cultures and settings.

Looking forward, technological innovations may dramatically expand how we understand and address loneliness in refugee populations. Digital phenotyping, the use of passive data from smartphones (e.g., movement, location, sleep, app usage, phone calls, SMS, social media patterns), can help identify individuals at high risk of loneliness or distress, as well as assess environmental factors, such as their exposure to news, advertisements, or even events like sirens or bombings.^7^ This enables tailored interventions aligned to behavioural patterns, cultural preferences, and moment-to-moment changes in mood or activity.^8^

Just-in-Time Adaptive Interventions (JITAIs), informed by real-time data from smartphones, can respond dynamically to users' needs.^9^ For example, if a person's phone usage suggests social withdrawal, the app might deliver a prompt to connect with others, suggest a walk, or offer a moment of guided reappraisal. These interventions maximize relevance and engagement while minimizing user burden.^9^

Large language models (LLMs), the engines behind advanced AI tools, could offer scalable, on-demand therapeutic support.^10^ Though still nascent in application to refugee health, LLMs could potentially serve as conversational agents that deliver psychoeducation 24/7, suggest coping strategies, or offer empathic support in a user's language, all in the absence of human clinicians. Ethical and data safeguards, cultural tailoring, and rigorous evaluation are essential, but the potential reach is considerable.

To close the research-to-practice gap, national and global refugee health strategies must prioritize loneliness not as a secondary concern but as a key determinant of health and well-being. Scalable digital interventions that address the emotional, cognitive, and social dimensions of loneliness offer a path forward.

As global displacement continues to rise, addressing loneliness among refugees is both a public health imperative and a moral one. We must meet this crisis not only with shelter and safety, but with tools that restore connection, agency, and dignity to those who have lost so much.

## Contributors

Conception: SL, FLA. Writing–first draft: SL, FLA. Review and Editing: all authors.

## Declaration of interests

The authors declare no competing interests.
